# Evaluation of Packaging Effects on the Phenolic Profile and Sensory Characteristics of Extra Virgin Olive Oil During Storage Using Liquid Chromatography Coupled with Mass Spectrometry

**DOI:** 10.3390/foods14142532

**Published:** 2025-07-19

**Authors:** Mohamed M. Abuhabib, Francesc M. Campins-Machado, Julián Lozano-Castellón, Antònia Ninot, Agustí Romero-Aroca, Rosa M. Lamuela-Raventós, Maria Pérez, Anna Vallverdú-Queralt

**Affiliations:** 1 Polyphenol Research Group, Department of Nutrition, Food Science and Gastronomy, Faculty of Pharmacy and Food Sciences, University of Barcelona, 08028 Barcelona, Spain; mohamed.abuhabib@ub.edu (M.M.A.); francesc.campins@ub.edu (F.M.C.-M.); jullozcas@gmail.com (J.L.-C.); lamuela@ub.edu (R.M.L.-R.); 2 Institute of Nutrition and Food Safety (INSA-UB), University of Barcelona, 08028 Barcelona, Spain; 3 CIBER Physiopathology of Obesity and Nutrition (CIBEROBN), Institute of Health Carlos III, 28029 Madrid, Spain; 4 Fruit Production Program, Institute of Agrifood Research and Technology (IRTA), Centre Mas Bové, 43120 Constantí, Spain; antonia.ninot@irta.cat (A.N.); agusti.romero@irta.cat (A.R.-A.)

**Keywords:** storage, packaging, phenolic compounds, sensory attributes, Corbella

## Abstract

The health benefits of extra virgin olive oil (EVOO), including improved cardiovascular health and metabolic function, are linked to its phenolic content. This study evaluated how storage duration and packaging affect the phenolic composition and sensory quality of Corbella EVOO. Oils were analyzed at production and after 6 and 12 months of storage in two types of packaging: bag-in-box; stainless steel containers with a nitrogen headspace. UPLC-MS/MS profiling quantified 23 phenolic compounds, predominantly secoiridoids such as oleuropein and ligstroside aglycones. Oleuropein aglycone increased over time, whereas ligstroside aglycone peaked mid-storage before declining, likely converting to oleocanthal. Lignans and flavonoids degraded during storage, although luteolin increased, potentially due to glucoside hydrolysis. Bag-in-box packaging better preserved phenolic content than stainless steel. A sensory analysis corroborated the chemical findings, with oils stored in stainless steel showing greater reductions in pungency and astringency. A Pearson correlation linked bitterness with oleuropein aglycone (r = 0.44) and oleacein (r = 0.66), pungency with oleocanthal (r = 0.81), and astringency with oleacein (r = 0.86) and oleocanthal (r = 0.71). These findings highlight the importance of packaging in preserving the phenolic composition responsible for the sensory qualities of EVOO over time.

## 1. Introduction

The Corbella olive, a traditional cultivar native to the Cardener Valley (Bages and Solsonès districts, Catalonia, Spain), has recently been revived and reintroduced into cultivation. This variety is now being grown in other regions of Catalonia, expanding its agricultural and economic significance [1]. Phenolic compounds are the key phytochemicals in extra virgin olive oil (EVOO), including secoiridoids, lignans, phenolic alcohols, phenolic acids, and flavonoids [1,2]. Among these, secoiridoids are the most abundant in fresh EVOO, primarily oleacein and oleocanthal, which are formed via the enzymatic hydrolysis of the glycosides oleuropein and ligstroside, respectively. Lignans (e.g., pinoresinol and 1-acetoxypinoresinol) represent the second major class of phenolic compounds, while flavonoids (e.g., luteolin, apigenin), phenolic acids (e.g., caffeic, ferulic, *p*-coumaric, and vanillic acids), and phenolic alcohols (e.g., hydroxytyrosol and tyrosol) contribute further to the bioactive profile of EVOO [3,4]. 

Liquid chromatography coupled with mass spectrometry (LC-MS) is a powerful and highly sensitive analytical technique widely used for the qualitative and quantitative analysis of phenolic compounds in EVOO [5,6]. This method offers excellent resolution and specificity, enabling the accurate identification of a broad range of phenolic constituents, including complex secoiridoids, lignans, flavonoids, and phenolic alcohols [7,8,9].

Storage conditions, including temperature, light exposure, duration, and packaging, play a critical role in preserving EVOO quality and determining its shelf-life [10,11]. Beside storage, environmental conditions, harvest, and production processes can affect the quality and quantity of olive oil [12]. Studies indicate that storage duration has a greater impact on the chemical composition of EVOO than agronomic or technological factors [9]. Additionally, contact with reactive materials, such as metal containers, must be avoided, as it can trigger oxidative degradation [13] and compromise oil stability. EVOO is commonly packaged in dark-colored glass, polyethylene terephthalate, tinplate, aluminum, plastic-coated paperboard (e.g., Tetra Brik), high-density polyethylene, and multilayer pouches (bag-in-box-type packaging) [14]. 

Pristouri et al. (2010) demonstrated that materials with high oxygen permeability, such as polypropylene and polyethylene, are unsuitable for olive oil storage [15]. They instead recommend using dark glass bottles stored below 22 °C and protected from light to preserve EVOO quality. More recent studies have explored the use of bag-in-box packaging for olive oil, revealing that while some phenolic degradation occurs over time, quality parameters remain within regulatory limits for EVOO. These findings suggest that the bag-in-box system is a viable option for maintaining olive oil quality, even under extreme temperature conditions [16,17].

To the best of our knowledge, no prior research has explored the combined effects of storage duration and packaging on the phenolic profile of the EVOO in relation to its sensory attributes. Therefore, the aim of this study was to evaluate the impact of storage time (6 and 12 months) and packaging type (bag-in-box; stainless steel containers with nitrogen (N_2_) in the headspace) on the stability and transformation of the main phenolic compounds in Corbella EVOO and their relationship with sensory quality. This assessment was performed through phenolic profiling and a sensory analysis. 

## 2. Materials and Methods

### 2.1. Reagents

Acetic acid, formic acid, methanol, acetonitrile (ACN), and *n*-hexane were from Sigma-Aldrich (Madrid, Spain). Regarding the standards (≥90% purity), oleocanthal was purchased from Merck (Darmstadt, Germany), and oleacein, oleuropein aglycone, and elenolic acid from Toronto Research Chemical Inc. (North York, ON, Canada). Oleuropein, ligstroside, and pinoresinol were acquired from Sigma-Aldrich (Darmstadt, Germany).

### 2.2. Olive Samples and Oil Production

Corbella olives were harvested by the company “AGRO-MIGJORN S.L.” on 6 and 13 October 2022, from an olive grove located in Barcelona, Catalonia, Spain (latitude 41°52′12.9″ N, longitude 1°44′35.9″ E; 400 m altitude, 87 km from Barcelona). The physical analysis of the olive fruits was performed either on the same day or the day after harvesting, following the parameters listed in Table S1. For the chemical analysis, the olives were immediately preserved at −80 °C. Prior to processing, the olives were cleaned and washed with water. The olives used for oil production had a maturity index ranging from 1.78 to 2.33, with individual weights between 1.30 and 1.46 g, and a moisture content of approximately 50%. The fat content ranged from 34.3% to 36.2%, and Brix values varied between 14.5% and 15.5%. All the physical parameters are presented in Table S2. Overall, all the samples were in good condition.

The oil was produced using the Oleomio 200 mill at AGRO MIGJORN S.L., following standard protocols. Key processing parameters were as follows: particle size: 6.5 mm; crushing speed: 10 L/h; malaxation temperature: 20 °C; malaxation time: 50 min. The solid–liquid and water–oil separation steps, as well as filtration, were performed according to the company’s standard protocol. Once extracted, the oil was transferred directly to two types of containers: bag-in-box; stainless steel containers filled with inert gas (N_2_). Both packaging systems were stored under controlled ambient conditions, with a temperature ranging between 22 and 23 °C. The oil was sampled at 6 and 12 months after production for analysis.

### 2.3. Prediction of Oil Shelf-Life

The estimation of the shelf-life was performed by three different methods described in Claudia Guillaume & Ravetti (2016) [18]:

- Method 1: Shelf-life (months) = Rancimat hours at 110 °C × 1.
- Method 2: Shelf-life (months) = [17.0% − Pyropheophytin *a* (PPPs)]/0.6%.
- Method 3: Shelf-life (months) = [1,2-Diacylglycerol Content (DAGs) − 35.0%]/Acidity factor (FFA factor).

FFA factor = 1.7% (if FFA < 0.4%); 2.1% (if 0.4% < FFA < 0.6%); or 2.5% (if FFA > 0.6%).

### 2.4. Phenolic Compound Analysis

The liquid–liquid extraction of phenolic compounds was performed following the method proposed by [19] with minor modifications. The entire extraction process was carried out on ice. Briefly, 0.5 g of EVOO was mixed with 5 mL of methanol in a 10 mL centrifuge tube and stirred for 30 s. The mixture was then centrifuged at 3000 rpm for 3 min at 4 °C. The methanolic phase was transferred into a clean flask and subjected to a second round of extraction. Both extracts were then pooled and concentrated under reduced pressure. The resulting residue was dissolved in 2 mL of acetonitrile and subsequently washed twice with 2 mL of hexane. Afterwards, the acetonitrile was removed by evaporation under vacuum, and the remaining residue was dissolved in 800 μL of a methanol–water solution (4:1, *v/v*). The solution was filtered using 0.2 μm polytetrafluoroethylene (PTFE) syringe filters, placed in amber glass vials, and stored at −80 °C until further analysis. 

The quantification of phenolic compounds was performed using liquid chromatography coupled to tandem mass spectrometry (LC-MS/MS), following the methodology previously described by our research group [2]. The equipment used was an Acquity TM UPLC system (Waters; Milford, MA, USA) coupled to an API 4000 triple-quadrupole mass spectrometer (PE Sciex, Concord, ON, Canada) with an ionic spray turbo as the ionization source, at the Separation Techniques Unit of the Scientific and Technological Centers (CCiTUB), Universitat de Barcelona. The column used was an Acquity UPLC^®^ (Uttar Pradesh, India) BEH C18 (2.1 × 50 mm, i.d., 1.7 μm particle size), and the pre-column was an Acquity UPLC^®^ BEH C18 (2.1 × 5 mm, i.d., 1.7 μm particle size) (Waters Corporation^®^, Wexford, Ireland). Two methods were employed:

Method A was used to determine the major secoiridoids (oleacein, oleocanthal, oleuropein aglycone, and ligstroside aglycone). The mobile phases were methanol (A) and water (B), both with 0.1% formic acid. The linear gradient (*v/v*) of A (t (min), %A) was as follows: (0, 5); (2, 5); (4, 100); (5, 100); (5.5, 5); (6.5, 5) [5].

Method B was used to determine the other phenolic compounds. The mobile phases were acetonitrile (A) and water with 0.05% acetic acid (B). The linear gradient (*v/v*) of A (t (min), %A) was as follows: (0, 2); (1, 2); (2, 5); (7.5, 40); (10.6, 60); (10.7, 100); (13.2, 100); (13.3, 2); (15, 2) [9]. The two methods were applied at a constant flow rate of 0.6 mL/min, an injection volume of 2 μL, and a column temperature of 50 °C. 

Ionization was in negative mode and with electrospray (ESI). All compounds were scanned by multi-reaction monitoring (MRM) with the parameters described in Table S3. The system software was ABSciex Analyst version 1.6.2, and the chromatograms were integrated using the same software. The quantification was performed with an external calibration curve using refined olive oil with the following standards: apigenin, hydroxytyrosol, pinoresinol, oleuropein, ligstroside, oleocanthal, oleacein, oleuropein aglycone, elenolic acid, and luteolin. Compounds lacking a commercial standard were quantified using structurally similar phenolic compounds.

### 2.5. Sensory Evaluation

The sensory analysis of the oil samples was performed by the Official Tasting Panel of Catalonia, following the regulations of the European Union (EU 2568/91, update) [20] and the International Olive Council (IOC/T.20 Doc. No. 15/Rev. 10/2018) [21]. The Catalonia Tasting Panel is officially recognized by both the EU and IOC and operates in compliance with ISO 17025, ISO 662, and ISO 659 standards [22,23,24]. Sensory profiling focused on the intensity of defects and three primary positive attributes: fruitiness, bitterness, and pungency. The tasting panel assessed each sample using a 10 cm intensity scale, where 0 cm indicated the absence of an attribute and 10 cm represented maximum intensity. Each taster individually evaluated the perceived intensity of both positive and negative attributes, and the final intensity score for each descriptor was calculated as the mean of evaluations from eight tasters. 

Secondary positive attributes, including astringency and green notes, were also evaluated, in accordance with IOC guidelines (2018). The complexity of sensory perceptions was assessed by analyzing the combination of positive attributes, with a greater number of perceived sensations indicating higher complexity. Samples were presented in randomized sequences during tasting sessions, with four samples evaluated per session. To ensure accuracy, ten-minute intervals were observed between sessions [21].

### 2.6. Statistical Analysis

All the experiments were performed in triplicate. Statistical analyses were conducted using the Metaboanlyst 6.0 (www.metaboanalyst.ca) online platform (accessed on 10 March 2025) [25], which employs the R-software program. A factorial analysis of variance (two-way ANOVA) was performed to assess the effects of storage time and packaging type on olive oil samples.

## 3. Results and Discussion

### 3.1. Oil Stability and Shelf-Life Analysis

The shelf-life of Corbella EVOO was evaluated using three predictive methods proposed by Guillaume and Ravetti (2016) [18], which are based on oxidative stability (Rancimat), pyropheophytin content (PPP), and 1,2-diacylglycerol levels (DAGs). These approaches provide a practical evaluation of the oil’s degradation state and its expected durability under standard storage conditions. The first method, based on Rancimat values at 110 °C, estimated a shelf-life of approximately 25.7 months. While using the PPP prediction method, the oil exhibited a projected shelf-life of over 27 months. The estimation of shelf-life based on the DAGs test indicated that the oil had an expected stability of over 34 months. These findings emphasize that the three quality tests used to predict EVOO shelf-life—each influenced by different factors over time—consistently confirm the remarkable stability and high quality of Corbella EVOO, with a minimum shelf-life exceeding two years.

### 3.2. Phenolic Profile Analysis

**Figure 1:**
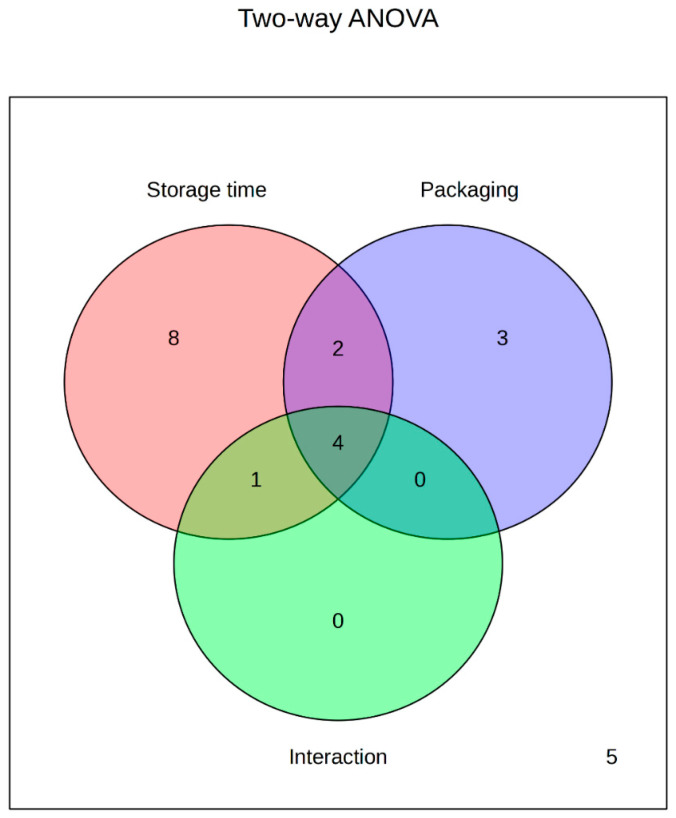
Diagram of factorial ANOVA results showing phenolic compounds affected by storage time and packaging type.

(Poly)phenolic compounds are naturally present in EVOO and play a crucial role in maintaining its chemical stability during storage. These bioactive constituents have attracted considerable scientific interest due to their antioxidant and free radical scavenging properties, which contribute both to EVOO shelf-life and to its potential health benefits [9,26]. In this study, a total of 23 phenolic compounds (Table 1), commonly found in EVOO, were quantified at the time of oil production and after six and twelve months of storage in two packaging systems: bag-in-box; stainless steel containers with a N_2_-filled headspace. The effects of storage time and packaging type—analyzed independently and interactively—on phenolic composition are presented in Table 2 and Figure 1.

#### 3.2.1. Effect of Storage Time on Polyphenols 

Secoiridoids constitute the most abundant class of phenolic compounds in EVOO and exhibit the highest transfer rate from olive fruits to oil. The major secoiridoids include oleuropein, ligstroside, and their derivatives [2,5]. The chemical structures of the main secoiridoids analyzed in this study are presented in Figure S1. During olive oil production and storage, these compounds undergo both enzymatic and non-enzymatic transformations, resulting in hydrolytic and oxidative modifications [27,28]. Hydrolysis, primarily catalyzed by β-glucosidase, cleaves the sugar moieties from oleuropein and ligstroside, yielding their respective aglycones [28].

Consistent with previous analyses of Corbella olives, which identified oleuropein aglycone—a hydrolytic product of oleuropein—as the major phenolic compound [1], our study confirmed its predominance in Corbella EVOO, with an initial concentration of approximately 51 mg/kg. This high level likely reflects elevated β-glucosidase activity in the olive fruit, promoting the hydrolysis of oleuropein into its aglycone form [2]. Over the 12-month storage period, oleuropein aglycone concentrations increased to 56 mg/kg in bag-in-box packaging and 67 mg/kg in stainless steel containers with a N_2_ headspace, likely due to continued hydrolysis over time.

Similarly to oleuropein aglycone, the concentration of ligstroside aglycone increased during the first six months of storage, rising from an initial 43.3 mg/kg to 57 mg/kg in the bag-in-box system and 60 mg/kg in the stainless steel containers with N_2_. This increase could be attributed to the enzymatic hydrolysis of ligstroside into its corresponding aglycone [28]. After 12 months, however, ligstroside aglycone levels declined to 45 mg/kg (bag-in-box) and 52 mg/kg (stainless steel with N_2_), suggesting progressive conversion to oleocanthal, as previously proposed [2,29]. These findings are consistent with earlier studies, which concluded that these secoiridoids initially accumulate during storage until degradation exceeds their formation [9]. In contrast, the concentration of oleocanthalic acid increased steadily in both packaging systems throughout the storage period, rising from 0.75 mg/kg to 0.95–0.98 mg/kg at six months and 1.77–1.81 mg/kg at 12 months. This increase likely results from the oxidative conversion of oleocanthal into its acid form, a process documented in the literature [30]. The slight increase in oleocanthalic acid concentration during storage in the stainless steel system suggests reduced protection against oxidation compared to the bag-in-box system.

Lignans represent the second most abundant class of phenolic compounds in EVOO after secoiridoids, with pinoresinol and lariciresinol being the predominant forms [31]. In our analysis, lariciresinol exhibited the highest initial concentration among lignans, at 15.4 mg/kg. However, after 12 months of storage, its concentration decreased significantly to 9.4 mg/kg in bag-in-box packaging and 12.3 mg/kg in stainless steel containers with a N_2_ headspace (Table 1). A similar trend was observed for pinoresinol, which decreased from 4.18 mg/kg to 3.76 mg/kg (bag-in-box) and 3.9 mg/kg (stainless steel with N_2_). This reduction in lignan content was likely due to enzymatic degradation, particularly through reductase activity [32].

In contrast, the two analyzed flavonoids, apigenin and luteolin, showed divergent trends over time. Apigenin exhibited a slight decrease, from 1.28 to approximately 1.2 mg/kg, over 12 months in both packaging systems (Table 1). The temporary undetectability of apigenin in the bag-in-box system at 6 months is likely due to analytical variability near the quantification limit, rather than a true absence, as apigenin typically occurs at low concentrations and may be influenced by matrix effects or transient ion suppression during LC-MS/MS analysis. Conversely, luteolin increased from below the quantification limit to over 3 mg/kg by the end of storage. This rise may be attributed to the hydrolysis of luteolin-*O*-glucoside, a compound known to be abundant in Corbella olives [1], yielding its aglycone form, luteolin.

#### 3.2.2. Effect of Packaging on (Poly) Phenolic Compounds

As a seasonal agricultural product, EVOO is produced within a limited timeframe but consumed throughout the year. Therefore, optimal packaging and storage conditions are essential for preserving its quality and health benefits [10]. Packaging materials play a crucial role in protecting EVOO from oxidative degradation, influencing both its chemical composition and shelf-life. As previously discussed, secoiridoids are the predominant phenolic compounds in EVOO, particularly oleuropein, ligstroside, and their derivatives [29]. Monitoring their hydrolysis and degradation provides insight into how packaging affects the quality of Corbella EVOO, especially during prolonged storage [2,16]. Our findings indicate that the packaging type had a more significant influence (*p* < 0.05) on phenolic compound degradation than storage duration (Table 2). After 12 months, oils stored in stainless steel containers with a N_2_ headspace showed higher levels of ligstroside aglycone (52 mg/kg) and oleocanthal (24.7 mg/kg) compared to those in bag-in-box packaging (45 and 17.9 mg/kg, respectively). A similar trend was observed for oleuropein aglycone and oleacein, with higher concentrations in stainless steel containers (67 and 53 mg/kg, respectively) than in the bag-in-box system (56 and 41.5 mg/kg, respectively) (Table 1). 

These findings suggest that polyphenol transformation is more pronounced in Corbella EVOO stored in stainless steel containers with nitrogen in the headspace compared to bag-in-box packaging. A previous study similarly found that bag-in-box packaging more effectively preserves the original high-quality phenolic profile of EVOO over time compared to tin-plated steel containers [16]. Dabbou et al. (2011) also concluded that packaging material has a stronger influence on olive oil stability than storage duration, emphasizing the importance of limiting both light and oxygen exposure to minimize oxidative degradation [33].

In summary, both storage duration and packaging type significantly altered the phenolic profile of EVOO. Olive oils stored in bag-in-box packaging exhibited less oxidation compared to those stored in stainless steel containers with a N_2_ headspace, particularly after 12 months. Although the use of N_2_ in stainless steel containers may provide some protection, it was less effective than the bag-in-box system in preserving oil quality over time. 

### 3.3. Sensory Analysis 

According to IOC standards, the classification of olive oils into categories (e.g., “extra virgin,” “virgin,” and “lampante”) is based on the assessment of both positive and negative sensory attributes [21]. To qualify as EVOO, the oil must exhibit clear fruity notes without any sensory defects. A sensory evaluation of the initial Corbella EVOO confirmed its classification as “extra virgin”, as it showed no sensory defects and had a fruitiness intensity of 4.9. The oil presented aromatic notes of freshly cut grass, with a moderate intensity of 3.6, and medium-intensity green fruitiness (Table 3). Regarding non-volatile attributes, which are closely linked to (poly)phenol content, the oil demonstrated high pungency (6.0) and bitterness (4.6), characteristic of a “robust” EVOO [34]. Regarding taste, the oil was distinctly spicy and bitter, with slight astringency and moderate sweetness. 

The lack of balance between these attributes could prove overwhelming for some consumers, as studies suggest that many prefer EVOOs with moderate bitterness and pungency [35]. Secondary aromas included notes of freshly cut grass and hints of almond and orchard plants, complemented by fresh fennel undertones. While the overall sensory profile of the oil may be perceived as overly robust by some, its green fruitiness and aromatic complexity contribute to its distinctive sensory appeal (Table 3).

**Figure 2:**
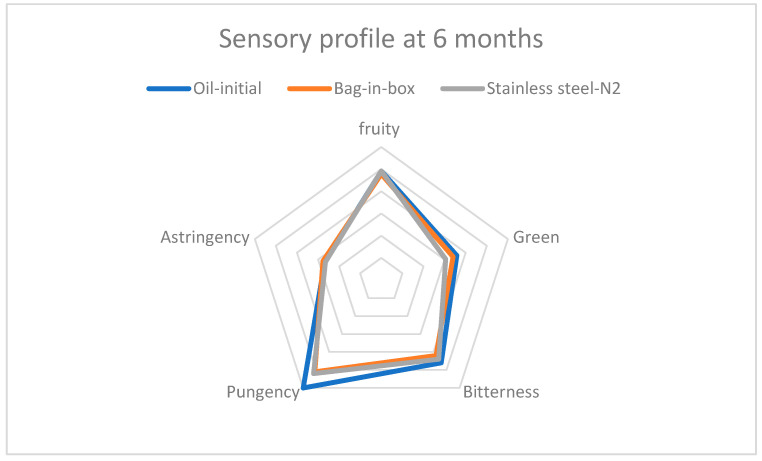
Sensory profile of the oil after 6 months of storage, according to packaging type.

**Figure 3:**
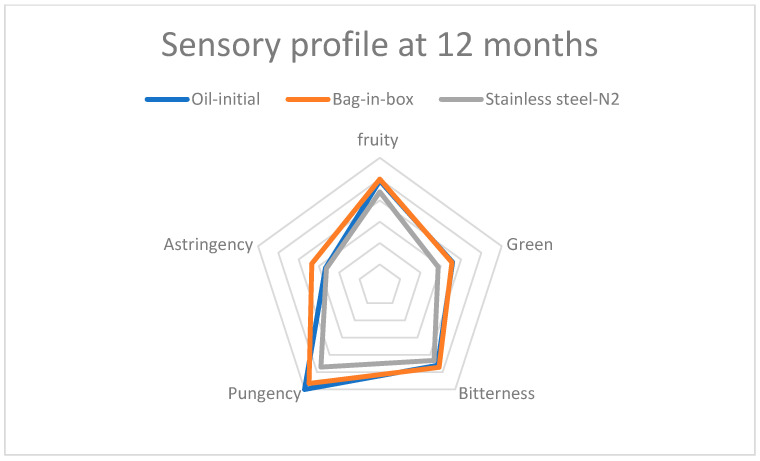
Sensory profile of the oil after 12 months of storage, according to packaging type.

A sensory evaluation at 6 and 12 months of storage revealed that Corbella EVOO retained its primary sensory characteristics—fruitiness, green aroma, bitterness, pungency, and astringency—with both packaging methods (Table 3). However, oils stored in stainless steel containers with N_2_ exhibited a significant decline in the intensity of all sensory attributes, particularly pungency and astringency (Figure 2 and Figure 3). In contrast, bag-in-box packaging better preserved sensory quality, with higher scores for all attributes. These results corroborate previous findings by Lolis et al. (2019) regarding the superior effectiveness of bag-in-box systems for olive oil storage [16].

**Figure 4:**
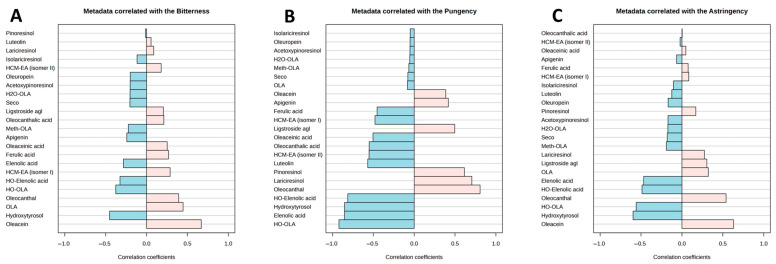
Pearson correlation coefficients between phenolic compound concentrations and sensory attributes: (**A**) bitterness, (**B**) pungency, and (**C**) astringency. HCM-EA: hydroxycarboxymethylated; H2O-OLA: hydro oleuropein aglycone; Seco: Secoisolariciresinol; Ligstroside agl: ligstroside aglycone; Meth-OLA: methyl oleuropein aglycone; HO-Elenolic acid: hydroxyelenolic acid; HO-OLA: hydroxy oleuropein aglycone; OLA: oleuropein aglycone.

A Pearson correlation analysis revealed a significant positive relationship between sensory attributes and phenolic compounds during storage. Bitterness correlated positively with oleacein (r = 0.66) and oleuropein aglycone (r = 0.44) (Figure 4A), supporting earlier findings that identified oleuropein aglycone as a key contributor to bitterness in virgin olive oil [36]. Pungency showed the strongest positive correlation with oleocanthal (r > 0.806) (Figure 4B), confirming its role as the principal compound responsible for the pungent sensation in EVOO [37]. Astringency was most strongly associated with oleacein (r = 0.86) and oleocanthal (r = 0.71), while showing moderate correlation with oleuropein aglycone (r = 0.59) (Figure 4C). These findings are consistent with previous reports indicating that oleuropein and ligstroside derivatives contribute significantly to astringency in olive oil [38]. However, oils stored in stainless steel containers with N_2_ exhibited higher aglycone concentrations (oleuropein aglycone, ligstroside aglycone, oleacein, and oleocanthal), while bitterness and astringency were unexpectedly lower. This apparent inconsistency can be attributed to the complex mechanisms underlying sensory perception. Genovese et al. (2020) demonstrated that while oleuropein and ligstroside aglycones strongly correlate with bitterness, their dialdehydic derivatives (oleocanthal and oleacein) are negatively associated with bitterness intensity [39,40]. Furthermore, the formation of phenolic-protein complexes in the oral cavity, driven by interactions with salivary proline-rich proteins and mucins, can reduce phenolic bioavailability and suppress sensory impact [41,42]. This supports our observation that elevated aglycones of the secoiridoids did not amplify bitterness or astringency in sensory testing.

## 4. Conclusions

This study demonstrates the effects of storage duration and packaging on both the phenolic profile and sensory characteristics of Corbella EVOO. The analysis confirmed that secoiridoids, mainly oleuropein aglycone and ligstroside aglycone, are the dominant phenolic compounds in Corbella EVOO and undergo significant transformations during storage. While the concentration of oleuropein aglycone increased progressively, ligstroside aglycone peaked mid-storage before declining, likely due to its conversion to oleocanthal. Lignans and flavonoids also showed time-dependent degradation: lignans decreased, while luteolin levels increased, attributable to glucoside hydrolysis. Although storage duration emerged as the primary factor influencing phenolic stability, packaging type also played a critical role. Stainless steel containers with nitrogen in the headspace preserved certain secoiridoids more effectively than bag-in-box packaging, but resulted in higher overall phenolic degradation. In contrast, bag-in-box packaging provided superior protection against oxidative degradation over 12 months. A sensory analysis reflected these chemical changes, with declines in pungency and astringency more evident in oil stored in stainless steel containers with nitrogen. Positive correlations were established between specific phenolic compounds and sensory attributes: oleuropein aglycone and oleacein with bitterness, oleocanthal with pungency, and both oleacein and oleocanthal with astringency. These findings confirm the functional role of phenolic compounds in defining the sensory quality of EVOO and the importance of selecting appropriate packaging to preserve oil during storage.

## Figures and Tables

**Table 1:** Phenolic compound concentration (mg/kg) in the initial oil sample and after 6 and 12 months of storage in bag-in-box and stainless steel containers.

		0 Months	6 Months		12 Months	
*Chemical Class*	Compound *	Initial Oil	Bag-in-Box	Stainless Steel with N_2_	Bag-in-Box	Stainless Steel with N_2_
*Secoiridoids*	Oleuropein	0.558 ± 0.005	ULQ	0.568 ± 0.004	0.560 ± 0.020	ULQ
	Oleuropein aglycone	51.000 ± 3.000	59.000 ± 4.000	64.000 ± 3.000	56.000 ± 2.000	67.000 ± 4.000
	Ligstroside aglycone	43.301 ± 1.700	57.000 ± 4.000	60.000 ± 3.000	45.000 ± 3.000	52.000 ± 2.000
	Oleacein	46.000 ± 2.000	44.000 ± 3.000	48.000 ± 2.000	41.500 ± 1.700	53.000 ± 3.000
	Oleocanthal	23.100 ± 1.300	24.300 ± 1.500	26.030 ± 1.120	17.900 ± 1.500	24.700 ± 1.400
	Oleacenic acid	0.569 ± 0.006	0.580 ± 0.015	0.592 ± 0.003	0.664 ± 0.016	0.686 ± 0.007
	Oleocanthalic acid	0.758 ± 0.019	0.950 ± 0.080	0.983 ± 0.019	1.770 ± 0.040	1.810 ± 0.060
	Hydroxy oleuropein aglycone	0.974 ± 0.017	1.030 ± 0.030	1.120 ± 0.040	1.520 ± 0.020	1.152 ± 0.016
	Hydro oleuropein aglycone	0.558 ± 0.005	ULQ	0.568 ± 0.004	0.560 ± 0.020	0.558 ± 0.002
	Methyl oleuropin aglycone	0.560 ± 0.005	ULQ	0.569 ± 0.005	0.560 ± 0.020	0.559 ± 0.002
	Hydroxyelolonolic acid	0.621 ± 0.016	0.613 ± 0.013	0.650 ± 0.030	0.710 ± 0.030	0.663 ± 0.009
*Secoiridoid derivatives*	Elenolic acid	3.500 ± 0.800	4.500 ± 1.200	5.200 ± 0.900	10.500 ± 0.300	6.200 ± 0.900
	HCM-EA (isomer I)	0.581 ± 0.006	0.600 ± 0.020	ULQ	0.748 ± 0.015	0.790 ± 0.007
	HCM-EA (Isomer II)	0.577 ± 0.005	0.595 ± 0.017	0.606 ± 0.002	0.660 ± 0.020	ULQ
*Phenolic alcohols*	Hydroxytyrosol	ULQ	ULQ	ULQ	0.120 ± 0.050	ULQ
*Flavonoids*	Apigenin	1.280 ± 0.03	ULQ	1.290 ± 0.020	1.190 ± 0.030	1.210 ± 0.040
	Luteolin	ULQ	2.604 ± 0.114	2.690 ± 0.170	3.060 ± 0.060	3.000 ± 0.200
*Phenolic acids*	Ferulic acid	ULQ	0.075 ± 0.009	ULQ	0.0920 ± 0.004	ULQ
*Lignans*	Pinoresinol	4.180 ± 0.090	4.200 ± 0.200	4.164 ± 0.105	3.760 ± 0.020	3.900 ± 0.080
	Acetoxypinoresinol	0.605 ± 0.005	0.604 ± 0.013	0.616 ± 0.005	0.600 ± 0.030	0.605 ± 0.002
	Lariciresinol	15.400 ± 1.400	13.900 ± 0.600	14.200 ± 0.700	9.400 ± 1.400	12.300 ± 1.300
	Isolariciresinol	3.700 ± 0.600	3.700 ± 0.400	4.500 ± 0.600	3.500 ± 0.400	4.200 ± 0.300
	Secoisolariciresinol	ULQ	0.156 ± 0.003	ULQ	0.155 ± 0.006	ULQ

* Quantification is expressed as mean ± standard deviation. ULQ: under the limit of quantification. HCM-EA: hydroxycarboxymethylated form of elenolic acid.

**Table 2:** Phenolic compounds with statistically significant changes (*p*-value < 0.05) in oil samples tested for stability.

*Chemical Class*	Compound	Storage Time	Packaging	Interaction: Storage Time–Packaging
*Secoiridoids*	Oleuropein			
	Oleuropein aglycone			
	Ligstroside aglycone	*	*	
	Oleacein		*	
	Oleocanthal	*	*	*
	Oleacenic acid	*	*	
	Oleocanthalic acid	*		
	Hydroxy oleuropein aglycone	*	*	*
	Hydro oleuropein aglycone			
	Methyl oleuropin aglycone			
	Hydroxyelolonolic acid	*		*
*Secoiridoid derivatives*	Elenolic acid	*	*	*
	HCM-EA (isomer I)	*	*	
	HCM-EA (Isomer II)	*		
*Phenolic alcohols*	Hydroxytyrosol	*	*	*
*Flavonoids*	Apigenin	*		
	Luteolin	*		
*Phenolic acids*	Ferulic acid	*		
*Lignans*	Pinoresinol	*		
	Acetoxypinoresinol			
	Lariciresinol		*	
	Isolariciresinol	*	*	
	Secoisolariciresinol			

* Differences due to this factor were significant. HCM-EA: hydroxycarboxymethylated form of elenolic acid.

**Table 3:** Average fruity, green, bitter, and pungent attributes of the oil at 0, 6, and 12 months.

	0 Months	6 Months		12 Months	
Attribute *	Initial Oil	Bag-in-Box	Stainless Steel with N_2_	Bag-in-Box	Stainless Steel with N_2_
Fruity	4.92 ± 0.51	4.78 ± 0.13	4.92 ± 0.30	5.00 ± 0.13	4.40 ± 0.44
Green	3.58 ± 0.45	3.40 ± 0.23	3.05 ± 0.48	3.55 ± 0.26	2.87 ± 0.15
Bitterness	4.60 ± 0.33	4.20 ± 0.23	4.40 ± 0.20	4.72 ± 0.12	4.32 ± 0.29
Pungency	6.00 ± 0.43	5.10 ± 0.13	5.20 ± 0.18	5.65 ± 0.10	4.70 ± 0.20
Astringency	2.68 ± 0.66	2.77 ± 0.13	2.65 ± 0.28	3.35 ± 0.44	2.63 ± 0.06

* Results are expressed as mean (of medians) ± standard deviation.

## Data Availability

The original contributions presented in the study are included in the article/Supplementary Materials, further inquiries can be directed to the corresponding authors.

## Supplementary Materials

The following supporting information can be downloaded at: https://www.mdpi.com/article/10.3390/foods14142532/s1, Table S1. Physical parameters of the fruits and methodologies used; Table S2. Physical characteristics of the samples of Corbella olive fruits; Table S3. Mass spectrometer parameters for each method used for quantification of the polyphenolic compounds; Figure S1. Chemical structures of the major secoiridoid compounds analyzed in Corbella EVOO.
