# Supplementary material for: Evaluation of Packaging Effects on the Phenolic Profile and Sensory Characteristics of Extra Virgin Olive Oil During Storage Using Liquid Chromatography Coupled with Mass Spectrometry
Source: Foods. 2025 Jul 19;14(14):2532. doi: 10.3390/foods14142532 (PMC12294515; doi:10.3390/foods14142532)
Supplement: Supplementary file 1 [file foods-14-02532-s001.zip › foods-3735434-supplementary.pdf]

Use of liquid chromatography coupled with mass spectrometry for the analysis of phenolic profile changes in extra virgin olive oil during storage and their sensory implications

Table S1. Physical parameters of the fruits and methodologies used.

Parameters	Methodology
Maturity Index (MI)	According to the method described by [1], it is a visual evaluation of the color of the fruit skin and pulp ranging from class 0, which corresponds to an intense green fruit color, to class 7, in which the fruit has a black skin and purple pulp down to the stone. The maturity index is the weighted average of a sample of 50 fruits of the different skin and flesh color classes.
Fruit weight	Gravimetry.
Pulp/stone ratio	Gravimetry.
Fruit moisture	Drying at 105 °C to constant weight (stove method; ISO 662) [23].
Fat	Soxhlet reference method (ISO 659) with VELP equipment, model SER158 on dried and crushed fruits [24].
Sugar content of olive juice (BRIX degrees)	Refractometry (ATAGO digital refractometer, Mod. PAL-1, Japan).
Health status	Visual detection (number of fruits with olive fly bites, fungi, or for some other biotic or abiotic reason that could condition the quality of the fruit).

Table S2. Physical characteristics of the samples of Corbella olive fruits.

Date of cultivation	* Maturity Index (MI)	Green index (%)	Health status (%healthy)	Fruit weight (g)	Pulp/Bone Ratio	Fruit moisture (%)	Fat content (%s.m.s)	Brix Degrees (%)
06/10/2022	1.78 ± 0.38	81 ± 4	60 ± 18	1.46 ± 0.23	1.91 ± 0.33	50.5 ± 2.4	36.1 ± 1.8	15.5 ± 0.6
13/10/2022	2.33 ± 0.40	55 ± 19	77± 6	1.30 ± 0.07	2.85 ± 1.02	50.0 ± 0.7	34.3 ± 1.2	14.5 ± 1.8

* Quantification is expressed as mean ± standard deviation.

Table S3. Mass spectrometer parameters for each method used for quantification of the polyphenolic compounds.

Parameters	Method A	Method B
Nebulizer Gas (NEB)	10 psi	12 psi
Gas curtain (CUR)	12 psi	12 psi
Collision Gas (CAD)	6	4
IonSpray Voltage (IS)	−4000 V	−3500 V
Temperature (TEM)	450 °C	500 °C
Collision Potential (CXP)	−15 V	−15 V

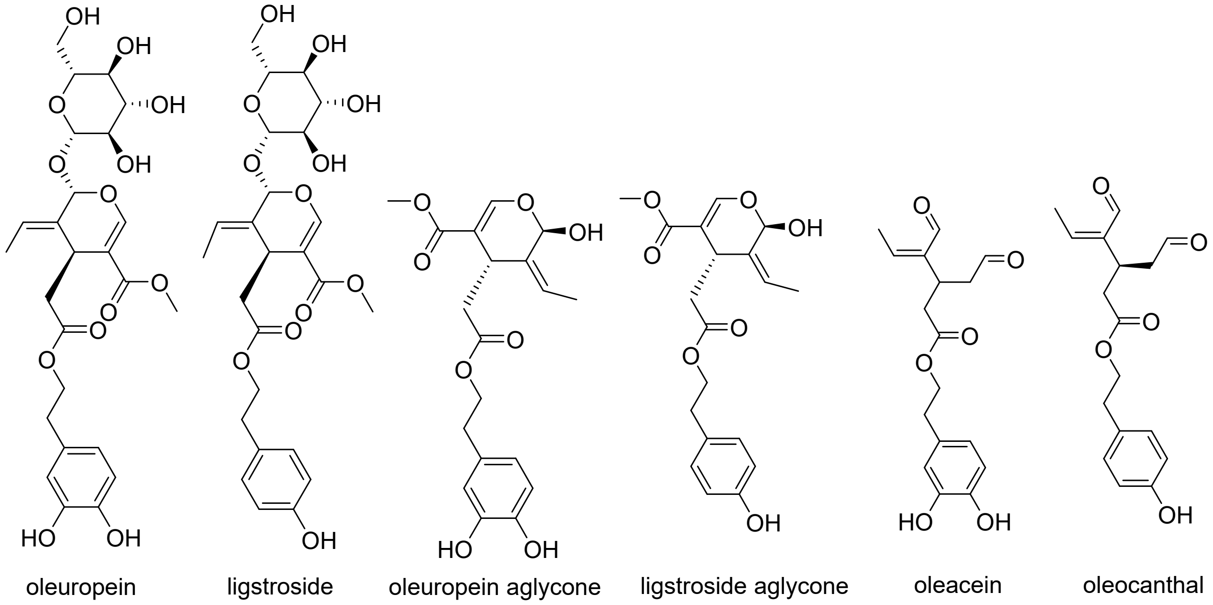

Figure S1. Chemical structures of the major secoiridoid compounds analyzed in Corbella EVOO.
